# Taxonomic study of *Baeosega* and its allies, with description of a new species of *Nipponosega* (Hymenoptera, Chrysididae, Amiseginae)

**DOI:** 10.3897/zookeys.1041.66267

**Published:** 2021-05-31

**Authors:** Toshiharu Mita

**Affiliations:** 1 Entomological Laboratory, Faculty of Agriculture, Kyushu University, Motooka 744, Nishi-ku, Fukuoka 819-0395, Japan Kyushu University Fukuoka Japan

**Keywords:** Asia, egg parasitoid, stick insects, synonymy

## Abstract

Three related genera of Asian Amiseginae, *Baeosega* Krombein, *Nipponosega* Kurzenko & Lelej, and *Okinawasega* Terayama are revised. The male of *N. yamanei* Kurzenko & Lelej and the female of *O. eguchii* Terayama are newly described. The following new synonymies are proposed: *Baeosega humida* Krombein, 1984 = *B. laticeps* Krombein, 1984, **syn. nov.**; *Nipponosega yamanei* Kurzenko & Lelej, 1994 = *N. kantoensis* Nagase, 1995, **syn. nov.** A new species of *Nipponosega*, *N. lineata***sp. nov.** is described based on a female from Thailand. A key to genera and species of *Baeosega*, *Nipponosega* and *Okinawasega* is given.

## Introduction

Amiseginae (Hymenoptera: Chrysididae) are egg parasitoids of stick insects (Phasmatodea) (Krombein 1983; Kimsey et al. 2013). They often show distinct sexual dimorphism: females are brachypterous; their wings are reduced to small pads reaching at most the anterior margin of the propodeum, whereas the males are macropterous. Because brachypterous females are seldom collected in the field and their morphological characters are greatly different from males, it is difficult to make correct sex association and thus many species have been known by only one sex (Kimsey and Bohart 1991; Kimsey et al. 2016).

Egg parasitoid wasps of stick insects are abundant in tropical and subtropical forests (Krombein 1983; Kimsey and Bohart 1991). However, some species occur in temperate regions. For example, Japan is known as the eastern and northern borders of their distribution in Asia and four genera have been recorded: *Nipponosega* Kurzenko & Lelej, 1994 (*N. yamanei* Kurzenko & Lelej, 1994 and *N. kantoensis* Nagase, 1995), *Cladobethylus* Kieffer, 1922 (*C. japonicus* Kimsey, 1986), *Calosega* Terayama, 1999 (*C. kamiteta* Terayama, 1999), and *Okinawasega* Terayama, 1999 (*O. eguchii* Terayama, 1999) (Kimsey 1986; Kurzenko and Lelej 1994; Nagase 1995; Terayama 1999). Except for *Calosega*, only the female or male of the species has been recognized. Although their life history is almost unknown, *N. yamanei* is considered to attack the eggs of *Micadina phluctainoides* (Rehn, 1904) (Diapheromeridae) (Mita 2014).

In the genus *Nipponosega*, three species are known from mainland China to Japan (Kurzenko and Lelej 1994; Nagase 1995; Xu et al. 2003). Although their males are unknown, the morphology of the female is closely related to *Baeosega* Krombein, 1983 and *Serendibula* Krombein, 1980. However, the female of *Serendibula* is clearly separated from *Baeosega* and *Nipponosega* by having a costate second metasomal tergite (Krombein 1983) and developed inner tooth of its claw. The monotypic genus *Okinawasega* was established on the basis of the male (Terayama 1999). The only known species, *O. eguchii* Terayama, endemic to southern Ryukyus, Japan, is apparently similar to the male of *Baeosega*. The genus has been insufficiently defined and thus it is difficult to distinguish it from *Baeosega* by the known diagnostic characters. Therefore, a comparative study of *Baeosega* and its allies is needed. *Baeosega* is known from three species from Sri Lanka (Krombein 1983) and Krombein (1983) mentioned the presence of a female of an undescribed species from Madras, southern India. In addition, Kimsey (1995) included southern Japan in its distribution, although species information was not provided. As for other related genera, the male of *Baeosega* is separated from *Serendibula*, the most similar taxon in Asia, by having longer setae on antennal flagellomeres, shorter metanotum (almost half as long as mesoscutum), and the absence of a developed inner tooth on the claw. For both genera, R1 on forewing is not indicated; however, unlike *Baeosega*, the distal apex of the pterostigma forms a sharp streak in *Serendibula*.

Although *Baeosega* is currently unknown outside of South Asia, the presence of *Nipponosega* and *Okinawasega* in East Asia suggests that other related taxa should be widely distributed. During the investigation of the Amiseginae fauna in Asia, several unknown females and males similar to *Baeosega* were found in Japan and Thailand. They provide helpful insights to understand taxonomic placement of the genera and species.

## Materials and methods

Examined materials are deposited in the collections of the following institutes:

**ELKU**Entomological Laboratory, Faculty of Agriculture, Kyushu University, Fukuoka, Japan;

**ELMU**Entomological Laboratory, Faculty of Agriculture, Meijo University, Nagoya, Japan (K. Yamagishi);

**MNHAH**Museum of Nature and Human Activities, Hyogo, Sanda, Japan (Y. Hashimoto);

**FFPRI** Forestry and Forest Products Research Institute, Tsukuba, Japan (S. Makino);

**NSMT** National Museum of Nature and Science, Tsukuba, Japan (T. Ide);

**THNHM** Natural History Museum of the National Science Museum, Thailand (W. Jaitrong);

**USNM**U.S. National Museum of Natural History, Smithsonian Institution, Washington D.C., U.S.A. (S. Brady).

Specimens were collected using flight interception traps (FIT), Malaise traps (MsT), yellow pan traps (YPT), emergence traps using leaf litter (EmT), leaf litter sifting, or net sweeping. Photos were obtained using an Olympus SZX10 stereomicroscope with an Olympus E-5 digital camera, or a Leica S8APO stereomicroscope with a Canon EOS Kiss X-5 digital camera. Images were combined using Zerene stacker ver. 1.04 (Zerene Systems, LLC, Richland, USA).

Morphological terminology follows that used by Krombein (1983) and Kimsey and Bohart (1991). The following abbreviations are used in the description:

**F1–F11** flagellomere numbers;

**MOD** anterior ocellus diameter;

**MS** maximum length of malar space;

**OL** distance between median ocellus and lateral ocellus;

**OOL** distance between lateral ocellus and compound eye;

**OPL** distance between lateral ocellus to posterior margin of vertex or occipital carina;

**POL** distance between lateral ocelli;

**T1–T3** metasomal tergite numbers.

Antennal articles were measured at the point of greatest breadth and compared with the total length of the article. The length of the pronotum was measured on the midline.

## Taxonomic account

### Family Chrysididae Latreille, 1802

#### Subfamily Amiseginae Mocsáry, 1889

### Key to the genera and species of *Baeosega* and its allies

Based on the key to genera of Amiseginae provided by Kimsey and Bohart (1991). Males of *Nipponosega kurzenkoi* and *N. lineata* sp. nov. are unknown.

**Table:** 

1	Micropterous (female)	**2**
–	Macropterous (male)	**7**
2	Deep malar sulcus present (Fig. 10D); mesosoma black (*Okinawasega*)	***O. eguchii* Terayama (Japan)**
–	Malar sulcus absent or only faintly indicated (Fig. 7C); mesosoma partly reddish or brownish	**3**
3	Occipital carina distinct from posterior margin of head to gena (Fig. 8C), rarely indistinct; head punctate with interspaces smooth (*Nipponosega*)	**4**
–	Occipital carina absent, at most posterior margin of vertex forming corner (Fig. 3C, D); head punctate with interspaces finely granulate (*Baeosega*)	**6**
4	Pronotum longitudinally costate (Fig. 7D)	***N. lineata* sp. nov. (Thailand)**
–	Pronotum sparsely punctate with smooth interspaces (Fig. 8E)	**5**
5	Mesopleuron fully testaceous; maximum interocular distance 1.2 × longer than width of frons	***N. kurzenkoi* Xu, He & Terayama (China)**
–	Mesopleuron at least partly blackish; maximum interocular distance 1.5–2.0 × longer than width of frons	***N. yamanei* Kurzenko & Lelej (Japan)**
6	Metasoma shagreened and dull (Fig. 5D); declivous anterior surface of T1 with weak longitudinal rugulae: silvery subdecumbent setae stout, conspicuous	***B. torrida* Krombein (Sri Lanka)**
–	Metasoma smooth, at most with faint granules; declivous anterior surface of T1 smooth; setae thinner, not conspicuous	***B. humida* Krombein, 1983 (Sri Lanka)**
7	F3 2.3 × as long as wide; and pronotum 0.7–0.9 × as long as mesoscutum (*Nipponosega*)	***N. yamanei* Kurzenko & Lelej**
–	F3 usually more than 3.5 ×, at least 2.7 × (*Baeosega humida*) as long as wide; and pronotum 1.0–1.1 × as long as mesoscutum	**8**
8	Forewing with R1 clearly indicated, linear (Fig. 11D); F3 3.8–4.3 × as long as wide; setae on flagellum 0.7 × as long as diameter of flagellomere (*Okinawasega*)	***O. eguchii* Terayama (Japan)**
–	Forewing with R1 not indicated (Fig. 4B); F3 2.7–3.5 × as long as wide; setae on flagellum 0.5 × as long as diameter of flagellomere (*Baeosega*)	**9**
9	Head and pronotum testaceous (Fig. 6); F3 3.5 × as long as wide	***B. torrida* Krombein (Sri Lanka)**
–	Dorsum of head blackish, pronotal disk reddish dark brown (Fig. 4); F3 2.7 × as long as wide	***B. humida* Krombein (Sri Lanka)**

#### Genus Baeosega

Taxon classificationAnimaliaHymenopteraChrysididae

Krombein

E13A5DAA-12CE-5A9D-8121-160F08082089

Baeosega Krombein, 1983. Type species: Baeosega torrida Krombein, 1983, original designation.

##### Diagnosis.

The female of *Baeosega* is superficially similar to *Nipponosega* and *Okinawasega*. However, the occipital carina is developed and reaching lower gena in *Nipponosega* (absent in *Baeosega*) and the deep malar sulcus is present in *Okinawasega* (only faintly indicated in *Baeosega*). The male is very similar to *Okinawasega*. The longer setae on flagellum and remarkably long R1 of the forewing are useful characters distinguishing *Okinawasega* from *Baeosega.* Compared to the above two genera, the male of *Nipponosega* has the pronotum short. The pronotum of *Baeosega* and *Okinawasega* is as long as or longer than mesoscutum but it is shorter in *Nipponosega*. Compared to genera found in South Asia, *Baeosega* is most similar to *Serendibula*. In the female of *Baeosega*, the metasomal T2 is lacking fine longitudinal carinae whereas T2 of *Serendibula* is covered with fine carinae. The male of *Baeosega* can be distinguished from *Serendibula* by having longer setae on antennal flagellomeres, the shorter metanotum, almost half as long as mesoscutellum (metanotum is longer, almost as long as mesoscutellum in *Serendibula*) and the tubular distal apex of pterostigma (very sharp in *Serendibula*). The inner tooth of tarsal claw is minute and indistinct in both female and male of *Baeosega*, whereas the inner tooth is distinctively large in *Serendibula*.

##### Description.

**Figure 1.**
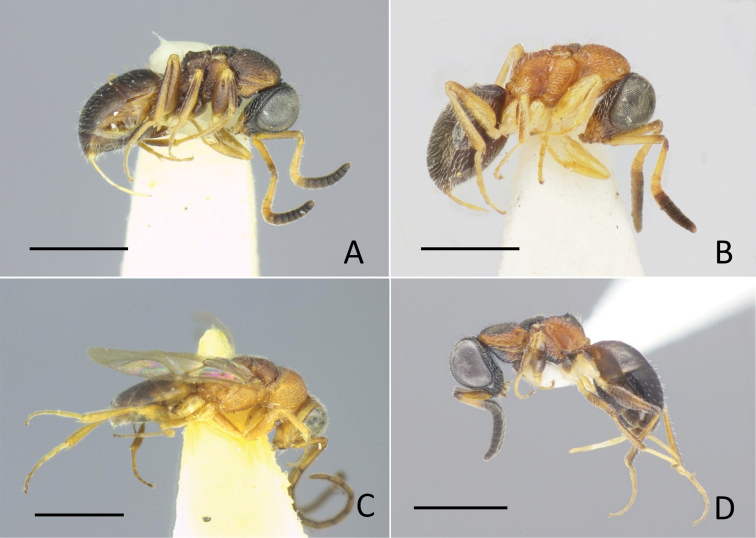
General habitus of Amiseginae**A***Baeosega humida* Krombein, holotype ♀ **B***B. torrida* Krombein, holotype ♀ **C** ditto, allotype ♂ **D***Nipponosega lineata* sp. nov., holotype ♀. Scale bars: 1.0 mm.

**Female.** Clypeal apex not thickened; malar sulcus absent or indicated as faint track; scapal basin shallow, cross-ridged, median longitudinal carina absent; occipital carina absent, at most posterior margin of vertex forming corner behind ocellar triangle; eye setose; flagellum fusiform, intermediate segments broader than long, and with ventral surface flattened. Mesosoma slender, dorsum more or less punctate; pronotum with median groove and shallow pit before lateral lobe, 1.0–1.4 × as long as combined length of mesoscutum, mesoscutellum and metanotum; mesoscutum with notauli; parapsides lacking; posterolateral corner of mesoscutum not lobate; micropterous (Fig. 1A, B), forewing pads extending to posterior margin of mesoscutellum; mesopleuron with omaulus, without scrobal sulcus; metanotum triangular and small, ca. 2/3 as long as mesoscutellum; propodeum with long dorsal surface and a pair of recumbent teeth present, meeting or almost meeting together, dorsal posterolateral angles bluntly angulate, lateral and posterior surfaces abruptly declivous. Hind coxa with dorsobasal carina; tarsal claws with a minute inner tooth. Metasoma smooth, shagreened or weakly granulated, without longitudinally striate area.

**Male.** Clypeal apex not thickened; scapal basin flat or weakly excavated, cross-ridged; malar sulcus present; occipital carina absent; eye setose; antenna elongate, F3 2.7–3.5 × longer than wide. Mesosoma slender, dorsum densely punctate; pronotum with median groove and shallow pit before lateral lobe, slightly longer than mesoscutum, 0.5–0.6 × as long as combined length of mesoscutum, mesoscutellum and metanotum; mesoscutum with notauli; parapsidal line faintly indicated; mesopleuron without omaulus and scrobal sulcus; metanotum approximately half mesoscutellum; a pair of recumbent teeth present, meeting or almost meeting together; propodeum with dorsal posterolateral angles bluntly angulate, posterior surface abruptly declivous; fully winged (Fig. 1C), pterostigma thin; R1 not indicated, distal apex tubular; Rs extended by weakly curved dark streak; medial vein arising before cu-a. Hind coxa with dorsobasal carina; tarsal claws with a minute inner tooth. Metasoma sparsely punctate with smooth interspaces.

##### Distribution.

Oriental region: Sri Lanka.

##### Hosts.

Unknown.

##### Remarks.

According to Krombein (1983), the female of *Baeosega* has no malar groove. However, a trace of a groove is present from the lower margin of the eye to the mandibular base. A minute inner tooth is also present in the claws of both female and male, but the size of tooth is remarkably small compared to *Serendibula*.

**Figure 2.**
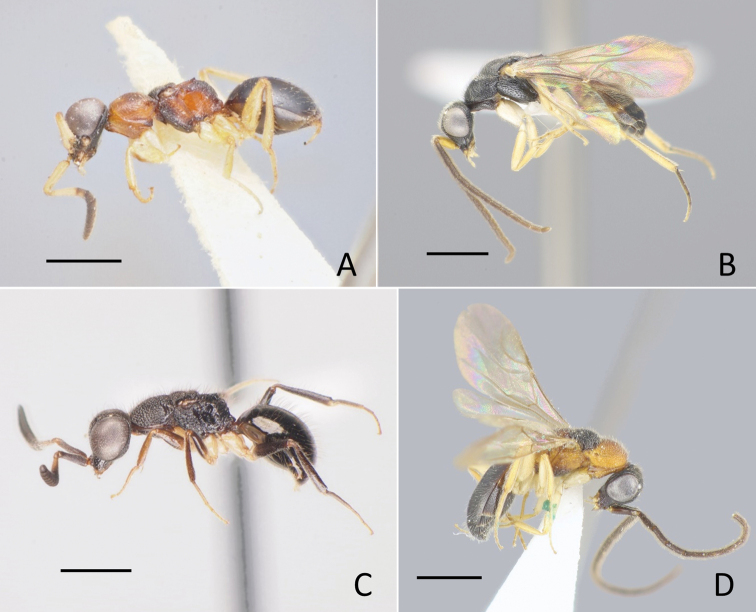
General habitus of Amiseginae**A***Nipponosega yamanei* Kurzenko & Lelej, holotype ♀ **B** ditto, ♂ **C***Okinawasega eguchii* Terayama ♀ **D** ditto, holotype ♂. Scale bars: 1.0 mm.

#### Baeosega humida

Taxon classificationAnimaliaHymenopteraChrysididae

Krombein, 1983

0D1BEE9A-C2F5-56A5-890B-4A03545B8995

Figures 1A , 3 , 4

Baeosega humida Krombein, 1983: 46, holotype ♀ by original designation. Type locality: Central Province, Kandy District, Kandy, Udawattakele Sanctuary (Sri Lanka).
Baeosega laticeps Krombein, 1983: 48, holotype ♀ by original designation, new synonymy. Type locality: Central Province, Kandy District, Kandy, Udawattakele Sanctuary (Sri Lanka).

##### Specimens examined.

***Holotypes*. *Baeosega humida***: Sri Lanka ♀, “SRI LANKA: Kan. Dist./ Udawattakele Sanct./ Elevation 1800 ft./ 23–25-IX-1980”, “Collected/ on or in/ leaf litter”, “K.V. Krombein/ P.B. Karunaratne/ L. Jayawickrema/ V. Gunawardane/ P. Liyanage” “HOLOTYPE/ BAEOSEGA/ HUMIDA/ Karl V. Krombein”, “Type No./ 100454/ U.S.N.M.” (USNM); ***Baeosega laticeps***: Sri Lanka ♀, “SRI LANKA: Kan. Dist./ Udawattakele Sanct./ 25–27-IV-1981”, “collected on or/ in leaf litter”, “K.V. Krombein/ T. Wijesinhe/ L. Jayawickrema”, “HOLOTYPE/ BAEOSEGA/ LATICEPS/ Karl V. Krombein”, “Type No./ 100455/ U.S.N.M.” (USNM). ***Paratypes*. *B. humida***: Sri Lanka 1♂ (allotype), same locality as holotype, but collected at 21–22.IX.1980 (USNM); 4♀, same as above but K.V. Krombein, L. Jayawickrema, V. Gunawardane, T. Wijesinhe leg. (USNM); 4♀1♂, same locality as above, but collected at 23–25.IX.1980, K.V. Krombein, P.B. Karunaratne, L. Jayawickrema, V. Gunawardane, P. Liyanage leg. (USNM); 1♀, same locality as above, but 12–14.X.1980, K.V. Krombein, P.B. Karunaratne, L. Jayawickrema, V. Gunawardane, T. Wijesinhe leg. (USNM); 8♀, same locality as above, but 25–27.IV.1981, K.V. Krombein, T. Wijesinhe, L. Weeratunge leg. (USNM); 1♂, same locality and collectors as above, but 22.V.1981 (USNM); ***B. laticeps***: Sri Lanka 2♀, same as locality as holotype of *B. laticeps*, but 12–14.X.1980, K.V. Krombein, P.B. Karunaratne, L. Jayawickrema, V. Gunawardane leg. (USNM).

##### Diagnosis.

**Figure 3.**
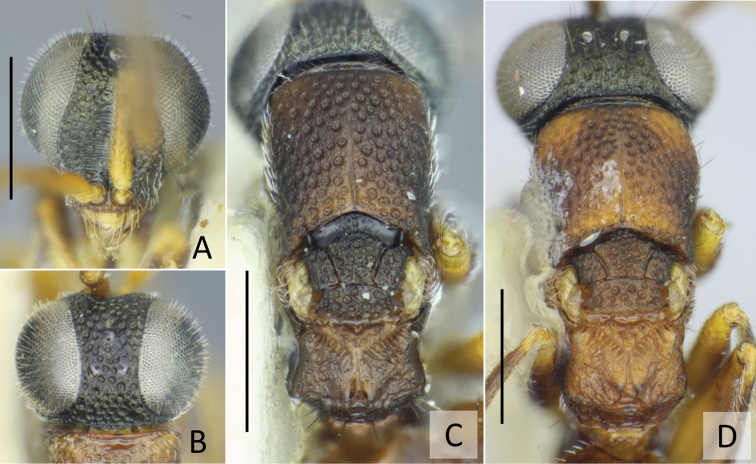
*Baeosega humida* Krombein ♀ **A** holotype of *B. humida*, head in frontal view **B** ditto, dorsal view **C** ditto, mesosoma **D** holotype of *B. laticeps*, mesosoma. Scale bars: 0.5 mm.

**Figure 4.**
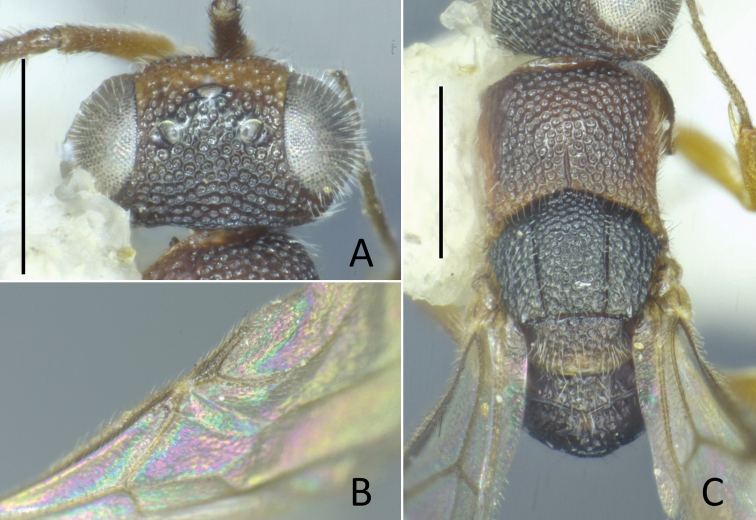
*Baeosega humida* Krombein, allotype ♂ **A** head in dorsal view **B** forewing **C** mesosoma. Scale bars: 0.5 mm.

The female of *Baeosega humida* Krombein can be distinguished from *B. torrida* Krombein, the other known species of *Baeosega*, by having the smooth metasoma. The male is similar to *B. torrida*, however, *B. humida* can be distinguished from *B. torrida* by the short F3 (2.7 × as long as wide). Punctures on mesepisternum are often separated with each other, but sometimes contiguous as in *B. torrida*. The body color of both female and male is brownish unlike *B. torrida*. Other diagnostic characters are as follows: (female) body length 2.3–2.7 mm; head (Figs 1A, 3A, B) black, bearing faint bronzy tint, punctate with interspaces granulated, except the costate scapal basin; malar sulcus indicated by a faint groove; occipital carina absent, only posterior margin of vertex forming corner behind ocellar triangle; mesosoma (Fig. 3C, D) dark brown to brown, part of pronotum, mesoscutum and metanotum darkened; surface punctate with interspaces granulated; (male) body length 2.3–2.7 mm; head (Fig. 4A) dorsally dark brown with face testaceous, punctate by densely located punctures; occipital carina absent; mesosoma (Fig. 4C) dark brown with mesoscutum blackish, punctate as head; postero-lateral corner of propodeum with distinctly producing angle.

##### Distribution.

Sri Lanka (Central Province, Kandy District).

##### Remarks.

According to Krombein (1983), the slender proportion of the head and mesosoma of *Baeosega laticeps* Krombein is the important diagnostic character separating it from *B. humida* Krombein. However, the body proportion can be variable to some extent. This variation is probably caused by the different shape of the host egg. Because there is no significant difference except for the proportion (slightly compressed laterally or not), *B. laticeps* is considered to be a junior subjective synonym of *B. humida*.

#### Baeosega torrida

Taxon classificationAnimaliaHymenopteraChrysididae

Krombein, 1983

CDA3FB12-6E83-58DD-BD1C-E0BB6572CFF4

**Figure 5.**
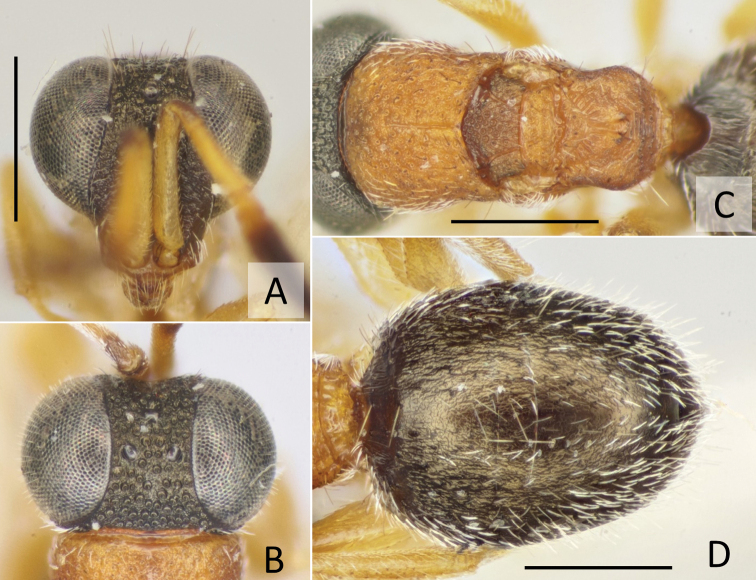
*Baeosega torrida* Krombein, holotype ♀ **A** head in frontal view **B** ditto, dorsal view **C** mesosoma **D** metasoma. Scale bars: 0.5 mm.

**Figure 6.**
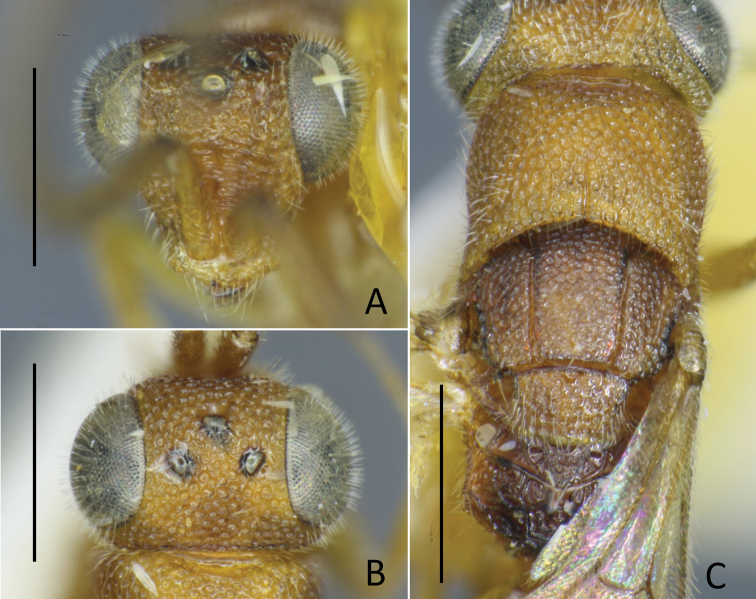
*Baeosega torrida* Krombein ♂ **A** head in frontal view, paratype **B** ditto, dorsal view, allotype **C** ditto, mesosoma. Scale bars: 0.5 mm.

Figures 1B, C , 5 , 6

Baeosega torrida Krombein, 1983: 44, holotype ♀ by original designation. Type locality: Angunakolapelessa, Uva District, Southern Province, Sri Lanka.

##### Specimens examined.

***Holotype*.** Sri Lanka ♀, “SRI LANKA: Mon. Dist./ Angunakolapelessa/ 22-28-III-1981”, “collected on or/ in leaf litter”, “K. V. Krombein/ T. Wijesinhe/ L. Weeratunge”, “HOLOTYPE/ BAEOSEGA/ TORRIDA/ Karl V. Krombein”, “Type No./ 100453/ U.S.N.M.” (hand-written) “2083468”. ***Paratypes*.** Sri Lanka 1♂ (allotype), same as holotype, but 100 m alt., 23.I.1979, MsT (USNM); 1♂, same as above (USNM).

##### Diagnosis.

The female of *Baeosega torrida* Krombein can be distinguished from *B. humida* Krombein by having the rugulose anterior declivity of T1 (Fig. 5C) and the strongly granulated dorsum of metasoma (Fig. 5D). Superficially, the reddish mesosoma is similar to *Nipponosega* females and *Serendibula deraniyagalai* Krombein. The male of *B. torrida* is similar to *B. humida*, however, the F3 is longer (3.5 × as long as wide) unlike *B. humida*. Other diagnostic characters are as follows: (female) body length 1.8–2.7 mm; head (Figs 1B, 5A, B) black, bearing faint bronzy tint, punctate with interspaces granulated; malar sulcus indicated by a faint groove; occipital carina absent; mesosoma (Figs 1B, 5C) punctate with interspaces strongly granulated; metasoma (Fig. 5D) blackish; stout silvery subdecumbent setae on mesosoma and metasoma; (male) body length 2.3–2.5 mm; head (Figs 1C, 6A, B) testaceous with dark flagellum, rarely around ocellar region brown, punctate by densely located punctures; occipital carina absent; mesosoma (Figs 1C, 6C) testaceous with mesoscutum, mesoscutellum, metanotum and dorsum of propodeum brown or rarely reddish, punctate as head; punctures on mesepisternum largely contiguous; postero-lateral corner of propodeum with weakly producing angle.

##### Distribution.

Sri Lanka (Uva Province: Monaragala District; Southern Province: Hambantota District; Central Province: Kandy District, Matale District)

#### Genus Nipponosega

Taxon classificationAnimaliaHymenopteraChrysididae

Kurzenko & Lelej

F09FDB86-D159-541F-9BB0-259760B2CD09

Nipponosega Kurzenko & Lelej, 1994: 83. Type species: Nipponosega yamanei Kurzenko & Lelej, 1994, original designation.

##### Diagnosis.

General characters of *Nipponosega* are similar to those of *Baeosega* and *Okinawasega*. The distinctive characters of *Nipponosega* are in the developed occipital carina in the female, the short setae on flagellum and the short pronotum in the male. More details, see the diagnosis of *Baeosega*.

##### Description.

**Female.** Clypeal apex not thickened; malar sulcus absent or indicated as faint track; scapal basin shallow, cross-ridged, median longitudinal smooth strip present; occipital carina present, reaching gena (Fig. 8C); eye setose; flagellum fusiform, intermediate segments broader than long, and with ventral surface flattened. Mesosoma slender, punctate or longitudinally striate; pronotum with median groove and shallow pit before lateral lobe, as long as combined length of mesoscutum, mesoscutellum and metanotum; mesoscutum with notauli and without parapsides; posterolateral corner of mesoscutum not lobate; micropterous (Figs 1D, 2A), forewing pads extending to posterior margin of mesoscutellum; mesopleuron with omaulus; scrobal sulcus lacking; metanotum triangular, longer than mesoscutellum; propodeum with long dorsal surface and a pair of recumbent teeth present, almost meeting together, dorsal posterolateral angles bluntly angulate, lateral surfaces abruptly declivous and posterior surface rounded. Hind coxa with dorsobasal carina; tarsal claws without inner tooth. Metasoma smooth.

**Male.** Clypeal apex not thickened; scapal basin flat, cross-ridged, median longitudinal smooth strip present; malar sulcus present; occipital carina absent (Fig. 9C); eye setose; antenna elongate, F3 2.3 × longer than wide. Mesosoma stout, dorsum punctate; pronotum with median groove and shallow pit before lateral lobe, 0.7–0.9 × as long as mesoscutum (Fig. 9E); median length of pronotum 0.4–0.5 × as long as combined length of mesoscutum, mesoscutellum and metanotum; mesoscutum with notauli; parapsidal line faintly indicated; mesopleuron without omaulus and scrobal sulcus; metanotum ca. 0.6 × as long as mesoscutellum; a pair of recumbent teeth present, slightly separated each other; propodeum with dorsal posterolateral angles rounded, posterior surface abruptly declivous; fully winged (Fig. 2B), R1 tubular, thick (Fig. 9D), not clearly differentiated from pterostigma; Rs extended by weakly curved dark streak; medial vein arising at or before cu-a. Hind coxa with dorsobasal carina; tarsal claws with one small inner tooth. Metasoma punctate with smooth interspaces.

##### Distribution.

Palaearctic region: Japan (Honshu, Shikoku, Kyushu, northern Ryukyus); Oriental region: China (Zhejiang); Thailand.

##### Hosts.

*Micadina phluctainoides* (Rehn, 1904) (Diapheromeridae) is considered to be the host of *Nipponosega yamanei* in Japan (Mita 2014).

#### Nipponosega kurzenkoi

Taxon classificationAnimaliaHymenopteraChrysididae

Xu, He & Terayama, 2003

F177DA38-7800-52F2-910D-BBE4865EAB2E

Nipponosega kurzenkoi Xu, He & Terayama, 2003: 195, holotype ♀ by original designation. Type locality: Mt. Jiulong, Suichang, Zhejiang Province, China (Type no. 944347, no type depository information, but likely Zhejiang University, Hangzhou). Not examined.

##### Diagnosis.

*Nipponosega kurzenkoi* is closely related to *N. yamanei* from Japan. It can be distinguished from *N. yamanei* by the fully testaceous mesopleuron, whereas it is blackish in *N. yamanei* (Fig. 2A). Also, the frons is wider than in *N. yamanei*. The maximum interocular distance (measured at the lower end of the face) is 1.2 × longer than the width of frons (1.5–2.0 in *N. yamanei*). Other diagnostic characters are as follows: body length 3.0 mm; head black, smooth with scattered punctures; lateral ocelli well separated from the inner margin of eye; mesosoma largely testaceous with mesoscutum and mesoscutellum black; pronotum smooth with scattered punctures; legs yellow; metasoma dark brown. Male is unknown.

##### Distribution.

China (Zhejiang).

##### Remarks.

Although the body color could be variable to some extent, no specimen of *N. yamanei* with completely testaceous mesopleuron has been found; a closely related species showing different distribution. The morphology of *N. kurzenkoi* should be evaluated in more detail to discuss their identification. However, diagnostic characters shown above do not overlap with those of *N. yamanei*. In the original description of *N. kurzenkoi*Xu et al., 2003, the evaluation of POD is different from the POD (= POL herein) used in Nagase (1995). The POD *sensu*Xu et al. (2003) is correspond to OOL herein.

#### Nipponosega lineata

Taxon classificationAnimaliaHymenopteraChrysididae

383462F5-06C1-5E4F-A16B-4B78EE50C5C8

http://zoobank.org/F30DAD50-8FC6-4A01-A304-8240DD8EC4F5

Figures 1D , 7

##### Specimen examined.

***Holotype*.** Thailand ♀ (THNHM-I-23985), “[NW Thailand] / Tak prov., / Umphang WS, / nr Pha Lueat stn.”, “28. i 2015 / Sk. Yamane leg. / 390 m alt.; leaf /& surface soil”, “Sk Yamane Collection” (THNHM).

##### Diagnosis.

**Figure 7.**
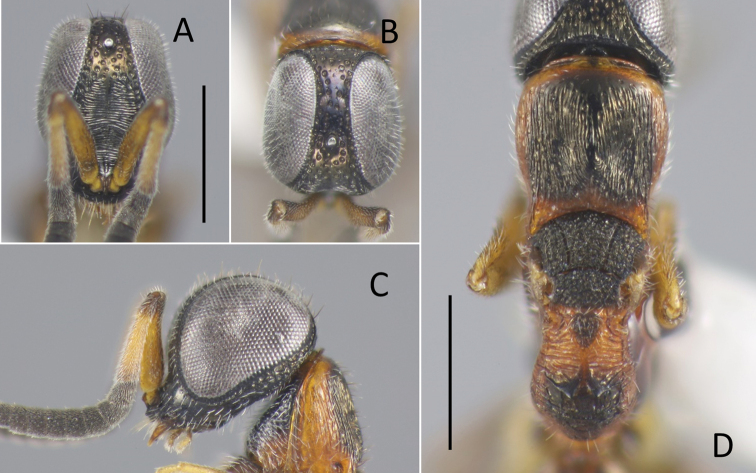
*Nipponosega lineata* sp. nov., holotype ♀ **A** head in frontal view **B** ditto, dorsal view **C** ditto, lateral view **D** vertex and mesosoma. Scale bars: 0.5 mm.

*Nipponosega lineata* sp. nov. is readily distinguished from other *Nipponosega* species by the longitudinally costate pronotum (Fig. 7D). Other characters useful to distinguish from other species are as follows: body length 2.8 mm; head black, punctate with interspaces smooth, posterior margin of vertex longitudinally costate (Fig. 7D); maximum interocular distance 2.9 × longer than width of frons; lateral ocelli almost touching the inner margin of eye; malar sulcus indicated as a faint track (Fig. 7C); outer orbit of eye with a row of punctures; occipital carina present (Fig. 7B, D), reaching lower gena; antenna blackish with scape and F1 testaceous; mesosoma reddish but dorsum largely black; mesoscutum and mesoscutellum punctate; legs brownish; metasoma dark brown, smooth. Male is unknown.

##### Description.

***Holotype* female.** Body length 2.81 mm. Head (Fig. 7A–C) punctate except scapal basin and posterior margin of vertex, as long as wide in dorsal view, 0.82 × as wide as deep in frontal view; punctures 0.5–1.0 × MOD, 0.5–2.0, 2.0–3.0, and, 0.5–1.0 punctures diameter apart on frons, ocellar region and vertex, respectively; interspaces among punctures smooth; scapal basin moderately excavated, transversely costate by fine grooves, upper part with short median unsculptured line; malar sulcus indicated as faint track; frons narrow, shortest distance between eyes 0.23 × head width; maximum interocular distance 2.9 × as long as narrowest width of frons; MS 0.23 × as long as eye height; outer orbit of eye with a row of deep punctures; ocellar triangle acute, lateral ocelli almost touching inner margin of eye; OL 1.3, OPL 2.2, POL 1.0, OOL 0.2, MOD 0.3; posterior margin of vertex longitudinally costate; occipital carina present, reaching gena. Clypeus thickened. Mandible without inner tooth, cylindrical. Antenna stout, F4–F9 wider than long; length (width) of F1 to F4 following ratio: 5.8 (1.5): 1.8 (1.7): 1.8 (1.8): 1.5 (2.0).

Pronotum (Fig. 7D) longitudinally costate by fine grooves except anterior narrow region smooth with several punctures; medial longitudinal line reaching 3/4 of pronotum; length of pronotum measured mesad 0.89 × as long as wide, 1.6 × mesoscutum plus mesoscutellum. Mesoscutum roughly punctate-reticulate: punctures as long as or slightly smaller than MOD; notauli complete, diverging anteriorly. Mesoscutellum punctate as mesoscutum, 0.59 × longer than mesoscutum. Mesopleuron punctate with smooth interspaces: punctures as long as MOD. Metanotum triangular, as long as mesoscutellum mesad, punctate. Propodeum (Fig. 7D) with postero-lateral corner rounded; dorsal surface long, smooth, transversely rugose; posterior surface transversely rugose; lateral surface obliquely rugose; metapleural region smooth but behind meso-metapleural suture costate. Middle and hind coxae transversely costate.

Metasoma smooth, covered with sparse setae; setae 2.5 × MOD.

***Color*.** Head black. Antenna basally testaceous with distal apex of scape and pedicel dark brown, F2–F10 black. Mandible brown. Mesosoma reddish but following parts blackish: dorsal surface of pronotum, anterior 2/3 of propleuron, mesoscutum, mesoscutellum, ventral surface of mesepisternum, median enclosure of metanotum, postero-dorsal surface of propodeum. Tegula and wings brown. Coxae whitish with postero-dorsal dark spot; trochanters testaceous; fore femur brown with basal and distal parts testaceous; middle and hind femora brown with basal parts testaceous; tibiae brown with basal parts of fore and hind tibiae testaceous; tarsi testaceous with fore and hind tarsi basally brownish. Metasoma dark brown with anterior surface of T1 and S1 paler.

##### Distribution.

Thailand (Tak Province).

##### Etymology.

The specific name derives from the Latin word *lineata*, referring to the presence of striae on the pronotum.

##### Remarks.

The holotype was collected from the leaf litter.

#### Nipponosega yamanei

Taxon classificationAnimaliaHymenopteraChrysididae

Kurzenko & Lelej, 1994

6CB2E38E-BEB1-535C-A863-3431E9A78536

**Figure 8.**
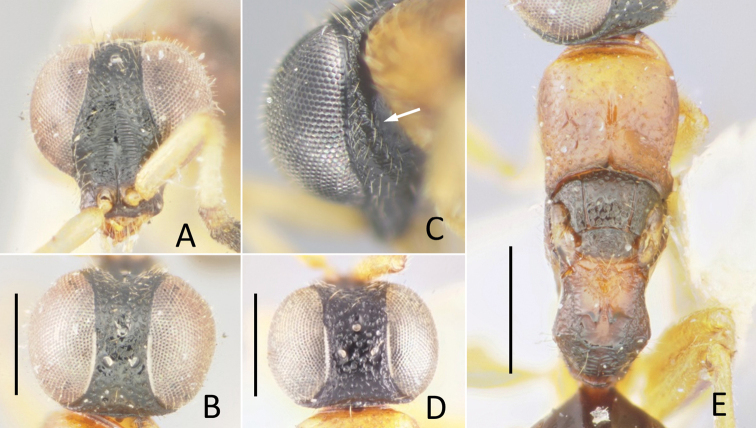
*Nipponosega yamanei* Kurzenko & Lelej ♀ **A** holotype of *N. yamanei*, head in frontal view **B** ditto, dorsal view **C** female from Aichi, gena (arrow indicates occipital carina) **D** holotype of *N. kantoensis* Nagase, head in dorsal view **E** holotype of *N. yamanei*, mesosoma. Scale bars: 0.5 mm.

**Figure 9.**
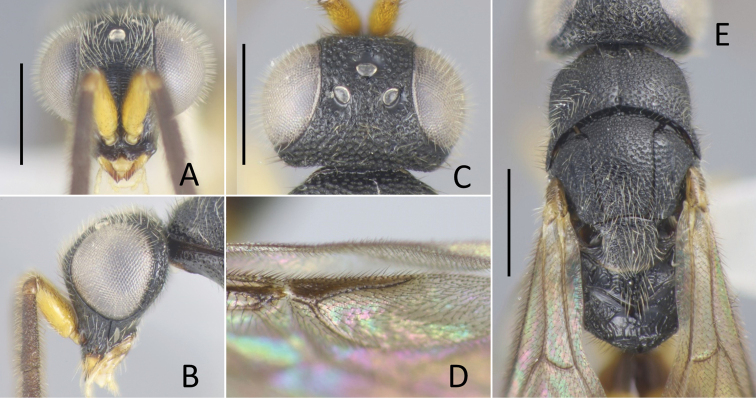
*Nipponosega yamanei* Kurzenko & Lelej ♂ **A** Head in frontal view **B** ditto, lateral view **C** ditto, dorsal view **D** forewing, pterostigma and R1 **E** mesosoma. Scale bars: 0.5 mm.

Figures 2A, B , 8 , 9

Nipponosega yamanei Kurzenko & Lelej, 1994: 83, holotype ♀ by original designation. Type locality: Shishitsuka Ohike, Tsuchiura City, Ibaraki Pref., Honshu (Japan).
Nipponosega kantoensis Nagase, 1995: 104, holotype ♀ by original designation, new synonymy. Type locality: Tonbo-Park, Sueno, Yoriimachi, Saitama Pref., Honshu (Japan).

##### Specimens examined.

***Holotypes*. *Nipponosega yamanei***: Japan – **Honshu** ♀, “JAPAN Ibaraki-ken / Shishitsuka Ohike / Tsuchiura City / Lelej 17 viii 1993”, “NSMT-HYM / 62329”, “Holotypus ♀ / *Nipponosega* / *yamanei* / Kurzenko et Lelej”(NSMT); ***Nipponosega kantoensis***: Japan ♀, “Tonbo-Park / Sueno, Yorii / Saitama / 11. VIII. 1991 / T. Nambu leg”, “NSMT-HYM / 62328”, “HOLOTYPE / *Nipponosega* / *kantoensis* / H. Nagase 1995”(NSMT).

***Other materials*.** Japan – **Honshu** 1♀2♂, Ogawa (600–800 m alt.), Kitaibaraki, Ibaraki Pref., 14–28.VIII.2002, MsT, H. Goto et al. leg. (ELKU); 1♂, same data, but (FFPRI); 3♀, Okami, Satomi-mura, Ibaraki Pref., 9–24.IX.2003, MsT, S. Makino et al. leg. (FFPRI); 1♀, Fujisawa-shi, Kanagawa Pref., 24–25.IX.2009, pit fall trap, T. Shimada leg. (ELKU); 1♂, Yawata (650 m alt.), Asahi, Aichi Pref., 29.VII–11.VIII.1998, MsT, A. Hanai, K. Yamagishi leg. (ELMU); 1♀, Yawata Shrine (400 m alt.), Asahi, Aichi Pref., 12–21.VIII.1998, MsT, M. Ozawa leg. (ELMU); 1♀, Kurisu, Inuyama, Aichi Pref., 19–25.VII.1996, EmT, T. Mabuchi leg.; 1♀, Mt. Sanage, Aichi Pref., 7–13.VIII.1992, EmT, K. Shima leg. (ELMU); 2♂, Takiwaki, Toyota, Aichi Pref., 8–14.VII.2002, MsT, Y. Kurahashi Leg. (ELMU); 1♀, same data, but 19–25.VIII.2002; 1♀, Tougoku, Seto, Aichi Pref., 10–16.VIII.1997, MsT, M. Kenmotsu leg. (ELMU); 1♀, Kurotani, Y., Hiroshima Pref., 7–13.VIII.1995, YPT, A. Morimoto leg. (ELMU); – **Shikoku** 1♀, Mt. Takanawa, Matsuyama, Ehime Pref., 26.VII.2017, K. Kuroda leg. (ELKU); 1♀, Jikiba-machi, Matsuyama-shi, Ehime Pref., 25.VII.2017, shifting, Y. Hisasue leg. (ELKU); – **Kyushu** 1♀, Mt. Kaya, Itoshima-shi, Fukuoka Pref., 2.X.2018, shifting, S. Inoue leg. (ELKU); 1♀, same locality as above, but Mt. Iwara, 8.IX.2019, K. Nishiya leg. (ELKU); 1♀, Hikosan Biological Laboratory, Kyushu University, Mt. Hiko (670 m alt.), Fukuoka Pref., 2.X.1968, MsT, K. Takeno leg.; 1♀, same data but 28–29.IX.1968; 1♀, same data but 15.X.1968; 1♂, same data but 4.IX.1970; 1♀, Mt. Hiko (700 m alt.), Fukuoka Pref., 20–28.VIII.2008, MsT, T. Mita, S. Sato leg. (ELKU); 1♀, Gokasho Plateau (860 m alt.), Takachiho-cho, Miyazaki Pref., 22.VIII.2015, Sk. Yamane leg. (ELKU); 2♀, same data but 23.VIII.2015; 1♀, Mt. Tatera, Tsushima Isl., Nagasaki Pref., 25–26. IX. 2015, YPT, Y. Hisasue leg. (ELKU); 1♀, same as above, but Mt. Shimizu, Izuhara-machi, 19.X.2019, T. Hashizume leg. (ELKU); – **Ryukyus** 2♀, Kurio, Yakushima Isl., 13.VII.1970, K. Yamagishi leg. (ELMU); 1♂, same island, but Miyanoura, 21.VI–11.VII.1999, MsT, T. Murota (A. Hanai) leg. (ELMU).

##### Diagnosis.

In the genus *Nipponosega*, *N. yamanei* Kurzenko & Lelej is the only species known by both female and male. The female is similar to *N. kurzenkoi* in China. They can be distinguished by the body coloration and the width of frons. The mesopleuron is blackish in *N. yamanei* (Fig. 2A), whereas it is fully testaceous in *N. kurzenkoi*. The frons is narrower, the maximum interocular distance is 1.5–2.0 × longer than the width of frons in full face view (1.2 in *N. kurzenkoi*). Other diagnostic characters of the female are as follows: body length 2.5–3.9 mm; head black, smooth with scattered punctures; posterior ocelli well separated from the inner margin of eye; pronotum reddish, smooth with scattered punctures; mesoscutum, mesoscutellum, anterior part of mesepisternum and often propodeum black; legs yellow; metasoma dark brown. The body length of the male is 3.2–3.8 mm. The male of other species of *Nipponosega* is unknown. Compared to other genera found in Japan, the male of *N. yamanei* can be distinguished by the head without occipital carina and impunctate welt; the body black, without metallic reflection; and the forewing with R1 thick, not clearly separated from pterostigma.

##### Description.

**Female**. Body length 2.5–3.9 mm. Head (Fig. 8A–D) punctate except scapal basin, 0.65–0.81 × as long as wide in dorsal view, 0.89–1.18 × as wide as deep in frontal view; punctures 0.3–0.5 × diameter of median ocellus, 0.3–1.0 punctures diameter apart on frons, 1.0–3.0 punctures diameter apart on ocellar region and vertex; interspaces among punctures smooth; scapal basin moderately excavated to almost flat, transversely costate by fine grooves, with median unsculptured line; malar sulcus absent; narrowest width of frons 0.24–0.33 × head width; maximum interocular distance 1.5–2.0 × as long as narrowest width of frons; MS 0.24–0.34 × as long as eye height; ocellar triangle variable, OL 0.7–1.6 × as long as POL, OL 0.8–1.2, OPL 2.0–2.5, POL 0.5–1.0, OOL 0.3–0.6, MOD 0.4–0.5; occipital carina present, reaching lower gena (Fig. 8C), occasionally present only behind ocellar triangle, only upper gena, or rarely almost invisible. Clypeus not thickened, with thin square transparent lobe. Mandible without inner tooth. Antenna stout, F2–F7 wider than long; length (width) of F1–F4 following ratio: 2.7–3.2 (1.0–1.2): 0.7–1.0 (1.0–1.3): 0.9–1.0 (1.1–1.3): 1.0 (1.1–1.4).

Pronotum (Fig. 8E) punctate with faintly coriaceous interspace; punctures slightly smaller than those on frons, 0.3–0.4 MOD, 3–5 PD apart; medial longitudinal line reaching 2/3 of pronotum, rarely groove fully developed; median length of pronotum 0.72–0.86 × as long as wide, 1.2–1.6 × mesoscutum plus mesoscutellum. Mesoscutum 0.43–0.50 × longer than pronotum, sculptured as vertex; notauli complete, diverging anteriorly. Mesoscutellum 0.5–0.75 × longer than mesoscutum, punctate as mesoscutum with interspaces faintly transversely rugulose. Mesopleuron sculptured as pronotum. Metanotum triangular, median length 0.67–0.87 × mesoscutellum, with shallow punctures. Propodeum with postero-lateral corner forming blunt angle (Fig. 8E), angle sometimes weak; dorsal surface of propodeum short, medially smooth, laterally rugose, sometimes irregularly reticulate; transverse or arched carina present behind; sometimes lateral carina present; posterior surface transversely costate with median longitudinal carina, sometimes laterally reticulate; lateral surface faintly costate with carinae antero-dorsally effaced; metapleural region polished or faintly transversely rugose. Middle and hind coxae transversely costate.

Metasoma faintly coriaceous, sparsely covered with setae; length of setae 2 MOD.

***Color*.** Head black. Antenna basally testaceous, F2–F10 dark brown, sometimes dorsal half of F2 testaceous. Mandible testaceous with reddish teeth. Mesosoma with prothorax, posterior half of mesopleuron and lateral surface of propodeum reddish to light brownish, remainder of mesothorax, metanotum and dorsal to posterior surface of propodeum brownish to blackish; dorsum of pronotum blackish in the female from Mt. Takanawa (Ehime Pref.); metanotum and propodeum sometimes paler especially in smaller specimens. Tegulae and wings testaceous to brown. Legs testaceous, sometimes femora and tibiae brownish. Metasoma brown to dark brown, rarely blackish, usually anterior surface of T1 and sterna paler.

**Male.** Body length 3.2–3.8 mm. Head (Fig. 9A–C) densely punctate except scapal basin, 0.5–0.57 × as long as wide in dorsal view, 1.16–1.46 × as wide as deep in frontal view; punctures 0.2–0.3 MOD, almost contiguous to 0.3 puncture diameter apart each; scapal basin excavated, transversely rugose with median unsculptured line; MS 0.3–0.4 × as long as eye height; narrowest width of frons 0.6–0.7 × head width; ocellar triangle obtuse, OL 0.5–0.6 × as long as POL, OL 0.8, OPL 2.0–2.8, POL 1.5, OOL 0.5–0.6, MOD 0.8–1.0. Clypeus not thickened, with thin transparent lamella. Mandible distally truncate. Antenna cylindrical, flagellum densely covered with setae; length of setae ca. 0.6 × flagellomere diameter; length (width) of F1 to F4 following ratio: 3.3–4.0 (0.8–1.0): 1.8–2.5 (0.8–1.1): 1.8–2.3 (0.8–1.0): 1.8–2.0 (0.8–1.0).

Pronotum (Fig. 9E) densely punctate as vertex; medial longitudinal groove present on posterior 3/5; median length of pronotum 0.46–0.58 × as long as wide, 0.7–0.9 × mesoscutum. Mesoscutum densely punctate as vertex; on posterior half interspaces faintly granulate; notauli complete, diverging anteriorly. Mesoscutellum punctate as mesoscutum, 0.45–0.63 × longer than mesoscutum. Mesopleuron punctate with polished interspaces; punctures slightly larger than those on pronotum, 0.3–1.5 puncture diameter apart; surface near pleural suture polished, without puncture. Metanotum with sparse punctures, median length 0.52–0.68 × mesoscutellum; punctures 1–3 puncture diameter apart. Propodeum postero-laterally forming dully corner, without teeth or distinct angle; dorsal surface more or less rugose, sometimes only median longitudinal carina present; transverse carina present between dorsal and posterior surface; posterior surface finely reticulate rugose with median longitudinal carina; lateral surface with ventral 2/3 finely reticulate, dorsal 1/3 smooth; metapleural region polished, more or less rugose and longitudinally excavated behind pleural suture. R1 tubular, thick, not clearly distinguished from pterostigma (Fig. 9D); Rs extended by weakly curved dark streak; medial vein arising at or before cu-a. Legs with surface of coxae smooth; hind coxa with dorsobasal carina; tarsal claws with one small inner tooth.

Dorsal surface of terga and sterna with fine punctures; punctures on T1 and T2 1–2 puncture diameters apart, with interspaces polished.

***Color*.** Head, mesosoma and metasoma black but anterior polished surface of T1 brownish, lateral surface of T1, T2 brown. Antenna with scape testaceous, flagellomere dark brown to brown, distally slightly paler. Mandible testaceous with apex reddish. Maxillary and labial palpus testaceous. Tegula brown. Wings faintly tinged with brown; veins brown. Legs testaceous with hind tibia dark brown.

##### Distribution.

Japan (Honshu; Shikoku; Kyushu; Tsushima Isl.; Yakushima Isl.).

##### Hosts.

Diapheromeridae: *Micadina phluctainoides* (Rehn, 1904).

##### Remarks.

Although ocelli and head proportions of the female have been considered useful for species classification (Nagase 1995), they actually show great variation. The holotypes of *N. yamanei* (Fig. 8B) and *N. kantoensis* (Fig. 8D) are examples of the two extremes and intermediate females are more frequently found. This makes it difficult to distinguish between the two types.

Males of *N. yamanei* were only obtained by Malaise traps and occasionally they were collected together with *Cladobethylus japonicus* Kimsey, 1997. The males of both species have been unknown; however, the morphological characters of the trapped males were close to those of *Baeosega*. As *Cladobethylus* is a genus showing little sexual dimorphism (Kimsey 2019), they are considered as *Nipponosega*. Compared to the abundance of the female, the male of *N. yamanei* is rarely collected. Some females were found above the ground surface (1 m or more) (Nagase 1995; Tomura 2020), but in fact they are more likely on the ground floor or in the leaf litter. Multiple females were obtained in Malaise traps, suggesting that females actively walk around the forest floor and climb understory vegetation. Females were observed to carry the host eggs for oviposition (Y. Hisasue, personal communication).

#### Genus Okinawasega

Taxon classificationAnimaliaHymenopteraChrysididae

Terayama

0C224E19-50AF-5C73-A50D-3DB05B211F27

Okinawasega Terayama, 1999: 99. Type species: Okinawasega eguchii Terayama, 1999, original designation.

##### Diagnosis.

General characters of *Okinawasega* are similar to those of *Baeosega* and *Nipponosega*; however, there are some distinctive differences, e.g., the deep malar sulcus in the female, the elongated linear R1 in the male. For more details, see the diagnosis of *Baeosega*.

##### Description.

**Figure 10.**
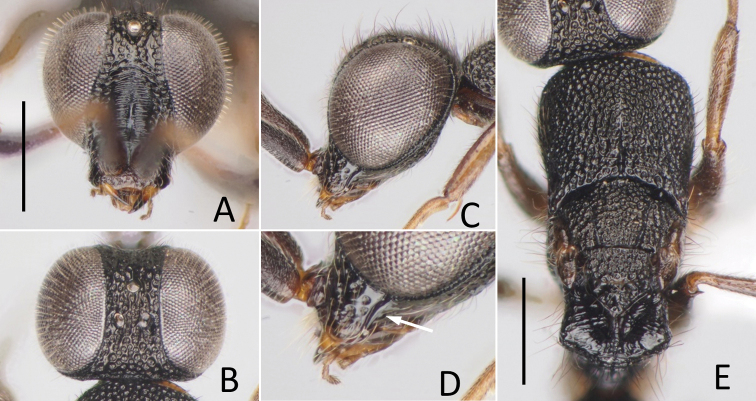
*Okinawasega eguchii* Terayama ♀ **A** head in frontal view **B** ditto, dorsal view **C** ditto, lateral view **D** ditto, malar space (arrow indicates malar sulcus) **E** mesosoma. Scale bars: 0.5 mm.

**Female.** Clypeal apex not thickened; malar sulcus present (Fig. 10D); scapal basin shallow, cross-ridged, median longitudinal carina present; occipital carina absent but posterior margin of vertex forming distinct corner behind ocellar triangle; eye setose; flagellum fusiform, intermediate segments broader than long, and with ventral surface flattened. Mesosoma slender, punctate by dense punctures; pronotum with median groove and shallow pit before lateral lobe, as long as combined length of mesoscutum, mesoscutellum and metanotum; mesoscutum with notauli and without parapsides; posterolateral corner of mesoscutum not lobate; micropterous (Fig. 2C), forewing pads extending to posterior margin of mesoscutellum; mesopleuron with omaulus, without scrobal sulcus; metanotum triangular and small, slightly shorter than mesoscutellum; propodeum with long dorsal surface and a pair of recumbent teeth present, almost meeting together, dorsal posterolateral angles bluntly angulate, lateral and posterior surfaces abruptly declivous. Hind coxa with dorsobasal carina; tarsal claws without inner tooth. Metasoma smooth.

**Figure 11.**
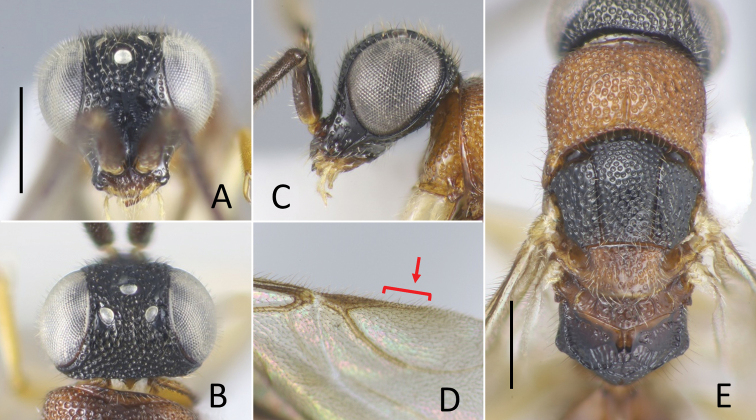
*Okinawasega eguchii* Terayama ♂ **A** head in frontal view **B** ditto, dorsal view **C** ditto, lateral view **D** forewing **E** mesosoma. Scale bars: 0.5 mm.

**Male.** Clypeal apex not thickened; scapal basin flat or weakly excavated, cross-ridged; malar sulcus present; occipital carina absent but posterior corner of vertex forming distinct corner behind ocellar triangle, occasionally trace of occipital carina present on upper gena; eye setose; antenna elongate, F3 3.5–4.3 × longer than wide. Mesosoma slender, dorsum punctate by dense punctures; pronotum with median groove and shallow pit before lateral lobe; pronotum as long as mesoscutum, 2/3 of combined length of mesoscutum, mesoscutellum; mesoscutum with notauli; parapsidal line faintly indicated; mesopleuron without omaulus and scrobal sulcus; metanotum approximately half mesoscutellum (Fig. 11E); a pair of recumbent teeth present, meeting or almost meeting together; propodeum with dorsal posterolateral angles bluntly angulate, posterior surface abruptly declivous; fully winged (Fig. 2D), pterostigma normal, with linear extension of R1 indicated, long (Fig. 11D, arrow); Rs extended by weakly curved dark streak; medial vein arising before cu-a. Hind coxa with dorsobasal carina; tarsal claws without inner tooth. Metasoma sparsely punctate with smooth interspaces.

##### Distribution.

Oriental region: Japan (Yaeyama Islands, southern Ryukyus).

##### Hosts.

Unknown.

##### Remarks.

The previous record of *Baeosega* in southern Ryukyus (Kimsey 1995) should probably be attributed to *Okinawasega*.

#### Okinawasega eguchii

Taxon classificationAnimaliaHymenopteraChrysididae

Terayama, 1999

55D25615-6E2E-5FC4-85ED-0BA7C9071163

Figures 2C, D , 10 , 11

Okinawasega eguchii Terayama, 1999: 100, holotype ♂, original designation. Type locality: Iriomote Island, Ryukyus, Japan.

##### Specimens examined.

***Holotype*.** Japan – **Ryukyus** ♂, “Holotype”, “*Okinawasega eguchii* Terayama, 1999”, ”Genotype *Okinawasega* Terayama, 1999”, “Iriomote-jima, Yaeyama Is., Okinawa Pref.”, “Japan”, “1. XI. 1995, K. Eguchi leg.”, “951101, Iriomote Is., Q1” (NMHAH).

***Other materials*.** Japan – **Ryukyus** 1♂, Shiramizu, Ishigaki Isl., 9.V.2004, T. Mita leg. (ELKU); 7♂, same data, but 10.V.2004 (ELKU); 4♂, same as above, but Mt. Omoto-dake, 15.V.2004, T. Tsuru leg. (ELKU); 3♂, Aira-gawa, Iriomote Isl., 8–12.X.2004, FIT, T. Ishikawa leg. (ELKU); 1♂, same data, but 14.V.2014, T. Mita leg. (ELKU); 17♂, same data, but 24–26.VI.2016, YPT, K. Komeda leg. (ELKU); 1♀24♂, same data, but 22–25.VI.2016 (ELKU); 1♀, same data, but 4.VII.2017, T. Kawano leg. (ELKU); 1♀, same island, but Mt. Tedou, 3.X.2017, K. Narita leg. (ELKU).

##### Diagnosis.

Conspicuous species in the southern Ryukyus (Japan). The female is blackish and covered with long setae, the body length is 3.5–3.6 mm. The male has long antennae and reddish body, the body length is 3.2–3.8 mm.

##### Description.

**Female.** Body length 3.5–3.6 mm. Head (Fig. 10A–D) densely punctate except scapal basin, 0.65–0.70 × as long as wide in dorsal view, 0.95–1.00 × as wide as deep in frontal view; punctures 0.3 MOD, almost contiguous but part of frons 1.0 puncture diameter apart each; scapal basin weakly excavated, transversely costate by fine grooves, with median unsculptured line; interspaces among punctures smooth; malar sulcus present (Fig. 10D); narrowest width of frons 0.3 × head width; MS 0.3 × as long as eye height; ocellar triangle acute, OL 1.0–1.2, OPL 2.3, POL 0.8–0.9, OOL 0.4–0.5, MOD 0.5–0.7; occipital carina absent but posterior margin of vertex forming distinct corner behind ocellar triangle. Clypeus not distinctly thickened, with thin square transparent lobe. Mandible without inner tooth. Antenna stout, F2–F9 wider than long; length (width) of F1 to F4 following ratio: 2.6–2.7 (1.1–1.2): 1.0 (1.3–1.4): 1.0 (1.4–1.5): 1.0 (1.5–1.7).

Pronotum (Fig. 10E) covered with densely located punctures, punctures same with those on head in size but deeper, slightly smaller mesad, somewhat longitudinally contiguous; medial longitudinal groove present on posterior 1/3 but polished strip leaching ca. 2/3; length of pronotum mesad 0.7–0.8 × as long as wide, 1.1–1.3 × mesoscutum plus mesoscutellum. Mesoscutum roughly puncto-reticulate, punctures 0.7 × larger than those on pronotum; notauli complete, diverging anteriorly. Mesoscutellum punctate as mesoscutum, 0.5–0.6 × longer than mesoscutum. Mesopleuron densely punctate; punctures larger, 0.7 MOD. Metanotum triangular, 0.7–0.9 × mesoscutellum mesad, punctate by shallow punctures. Propodeum with dorsal, posterior and lateral surface transversely rugose by obscure striae; median longitudinal carina present; metapleural region polished, finely rugose behind meso-metapleural suture, carinated above metacoxa; postero-lateral corner forming dully angle.

Metasoma smooth, sparsely covered with long setae; setae 3 × longer than MOD.

***Color*.** Head, mesosoma and metasoma black but lateral surface of T1 brown, S1 dark brown. Antenna black but scape dark brown, pedicel and F1 brown. Mandible pale brown with apex black. Maxillary and labial palpi brown. Tegula and wings dark brown. Coxae and trochanters testaceous; remainder of fore and middle legs brown; remainder of hind leg dark brown.

**Male.** Body length 3.6–3.7 mm. Head (Fig. 11A–C) densely punctate except scapal basin, 0.59–0.66 × as long as wide in dorsal view, 1.14–1.21 × as wide as deep in frontal view; punctures 0.2–0.3 MOD, interspaces ca. 0.3–1.0 puncture diameter; scapal basin faintly excavated, transversely rugose with median unsculptured line; MS 0.4–0.5 × as long as eye height; narrowest width of frons 0.63–0.76 × head width; ocellar triangle obtuse, OL 0.8–1.0, OPL 2.3–3.0, POL 1.3–1.5, OOL 0.05, MOD 0.8; occipital carina absent but posterior corner of vertex forming distinct corner behind ocellar triangle, occasionally trace of occipital carina present on upper gena. Clypeus thin. Mandible distally truncate. Antenna cylindrical, flagellum densely covered with long setae; length of setae ca. 0.8 × flagellomere diameter; length (width) of F1 to F4 following ratio: 4.3–4.7 (0.7–1.0): 3.0 (0.7–1.0): 3.0 (0.7–0.8): 2.8–3.0 (0.7–0.8).

Pronotum (Fig. 11E) punctate as vertex; medial longitudinal groove variable, present posterior 1/3 to reaching anterior margin of disc; length of pronotum mesad 0.7–0.8 × as long as wide, 0.9–1.1 × mesoscutum. Mesoscutum punctate as head, but punctures slightly smaller than those on vertex, 0.2–0.5 puncture diameter apart; notauli complete, diverging anteriorly. Mesoscutellum scattered with shallow punctures with polished interspace, 0.50–0.58 × longer than mesoscutum. Mesopleuron punctate with polished interspace; punctures slightly larger than those on pronotum, 0.3–1.0 puncture diameter apart; subalar fossa indicated by continuous row of punctures or groove, rarely absent; usually surface near pleural suture polished, without punctures. Metanotum 0.48–0.60 × mesoscutellum, punctate as mesoscutellum. Propodeum (Fig. 11E) postero-laterally forming dull corner; dorsal surface reticulate-rugose with median and usually two pairs of longitudinal carinae present; transverse carina present between dorsal and posterior surface; posterior and lateral surface reticulate-rugose; median longitudinal carina present on posterior surface; metapleural region polished, more or less rugose and longitudinally excavated behind meso-metapleural suture, carinated above hind coxa. Pterostigma narrow, with linear extension of R1 indicated, long (Fig. 11D, arrow); Rs extended by weakly curved dark streak; medial vein arising before cu-a. Hind coxa with dorsobasal carina; tarsal claws without inner tooth.

***Color*.** Head black. Antenna dark brown, rarely blackish. Mandible testaceous with apex dark brown. Maxillary and labial palpus testaceous. Mesosoma mostly reddish to dark reddish except mesoscutum black, tegula brown, propodeum with posterior surface darker, rarely entirely blackish; rarely dorsum of mesosoma blackish. Wings faintly tinged with brown; veins brown, rarely dark brown. Legs testaceous. Metasoma blackish except anterior polished surface of T1 brownish.

##### Distribution.

Japan: Yaeyama Islands, Southern Ryukyus (Ishigaki Isl., Iriomote Isl.)

##### Remarks.

No species in *Baeosega*-related genera are known from Ishigaki-jima and Iriomote-jima except for the male of *Okinawasega eguchii* Terayama. The newly found female was clearly related to *Baeosega*, and was therefore assigned to *Okinawasega*. Compared to males, females are seldom collected. The females actively walk on the ground surface and sometimes leap a small distance.

## Discussion

The discovery of previously unknown sexes of *Nipponosega* and *Okinawasega* revealed that the male morphology of the two genera and *Baeosega* is rather conservative, even though females show clear differences. Males share following features: the absence of the occipital carina; mesopleuron without omaulus and scrobal sulcus; the relatively shorter metanotum, which is 0.5–0.7 × as long as mesoscutellum; the tarsal claw without large inner tooth, at most one small tooth. As for the posterior margin of the head in females, an indistinct occipital carina is sometimes present behind the ocellar triangle in *Baeosega* and *Okinawasega*. However, the structure is not clearly cariniform. It is observed only as faintly suppressed posterior margin (Fig. 3C). The distinct occipital carina is present only in the female of *Nipponosega* (Figs 7D, 8C). In males, some genera have the forewing with R1 distinguishable from the distal part of pterostigma (e.g., *Imasega* Krombein and *Mahinda* Krombein). This linear extension of R1 is not indicated in *Baeosega* (Fig. 4B) and *Nipponosega* (Fig. 9D) but a long linear R1 is clearly indicated in *Okinawasega* (Fig. 11D). While further research is required, the R1 condition can be a diagnostic character of amisegine genera (Krombein 1983; Kimsey and Bohart 1991).

In *B. humida* and *N. yamanei*, the body proportions of females can vary within a species. Although only a few records on the host of Amiseginae are available, the adult body size of egg parasitoids is usually affected by the host, for example, the size and age of the host egg (Da Rocha et al. 2007). Additionally, discontinuous differences can be observed in egg parasitoids if they attack different host eggs, as is known in Scelionidae (Arakawa et al. 2004; Abram et al. 2016; Botch and Delfosse 2018). Further studies on their host and their life history will provide a clearer picture of the background of the host-dependent variation.

## Supplementary Material

XML Treatment for Baeosega

XML Treatment for Baeosega humida

XML Treatment for Baeosega torrida

XML Treatment for Nipponosega

XML Treatment for Nipponosega kurzenkoi

XML Treatment for Nipponosega lineata

XML Treatment for Nipponosega yamanei

XML Treatment for Okinawasega

XML Treatment for Okinawasega eguchii

## References

[B1] Abram PK Parent J-P Brodeur J Boivin G (2016) Size-induced phenotypic reaction norms in a parasitoid wasp: an examination of life-history and behavioural traits. Biological Journal of the Linnean Society 117: 620–632. 10.1111/bij.12658

[B2] Arakawa R Miura M Fujita M (2004) Effects of host species on the body size, fecundity, and longevity of *Trissolcus mitsukurii* (Hymenoptera: Scelionidae), a solitary egg parasitoid of stink bugs. Applied Entomology and Zoology 39: 177–181. 10.1303/aez.2004.177

[B3] Botch PS Delfosse ES (2018) Host-acceptance behavior of *Trissolcus japonicus* (Hymenoptera: Scelionidae) reared on the invasive *Halyomorpha halys* (Heteroptera: Pentatomidae) and nontarget species. Environmental Entomology 47: 403–411. 10.1093/ee/nvy014 29506058

[B4] Da Rocha L Kolberg R de Mendonça MS Redaelli LR (2007) Body size variation in *Gryon gallardoi* related to age and size of the host. BioControl 52: 161–173. 10.1007/s10526-006-9024-6

[B5] Kimsey LS (1986) New species and genera of Amiseginae from Asia (Chrysididae, Hymenoptera). Psyche 93: 153–165. 10.1155/1986/31631

[B6] Kimsey LS (1995) New amisegine wasps from Southeast Asia (Hymenoptera: Chrysididae). Entomological Society of Washington 97: 590–595.

[B7] Kimsey LS (2019) Revision of the south Asian amisegine genus *Cladobethylus* Kieffer, 1922 (Hymenoptera, Chrysididae, Amiseginae). Journal of Hymenoptera Research 70: 41–64. 10.3897/jhr.70.34206

[B8] Kimsey LS Bohart RM (1991 [1990]) The chrysidid wasps of the world. Oxford Science Publications, New York, 652 pp.

[B9] Kimsey LS Dewhurst CF Nyaure S (2013) New species of egg parasites from the Oil Palm Stick Insect (*Eurycantha insularis*) in Papua New Guinea (Hymenoptera, Chrysididae, Phasmatodea, Phasmatidae). Journal of Hymenoptera Research 30: 19–28. 10.3897/jhr.30.4010

[B10] Kimsey LS Mita T Pham HT (2016) New species of the genus *Mahinda* Krombein, 1983 (Hymenoptera, Chrysididae, Amiseginae). ZooKeys 551: 145–154. 10.3897/zookeys.551.6168 PMC4741294 26877668

[B11] Krombein KV (1983) Biosystematic studies of Ceylonese wasps, XI: a monograph of the Amiseginae and Loboscelidiinae (Hymenoptera: Chrysididae). Journal of the Kansas Entomological Society (376): 1–79. 10.5479/si.00810282.376

[B12] Kurzenko NV Lelej AS (1994) *Nipponosega yamanei* gen. et sp. nov., a new remarkable Cuckoo Wasp (Hymenoptera, Chrysididae, Amiseginae) from Japan. Bulletin of the National Science Museum. Series A, Zoology 20: 83–86.

[B13] Mita T (2014) Bees and wasps. In: Okajima S (Ed.) Pictorial book of Gakken, Live, Insects.Gakken Plus, Tokyo, 134–146. [in Japanese]

[B14] Nagase H (1995) A new species of *Nipponosega* (Hymenoptera, Chrysididae, Amiseginae) from central Japan. Bulletin of the National Science Museum. Series A, Zoology 21: 103–107.

[B15] Terayama M (1999) Descriptions of new species and genera of the Chrysidoidea (Insecta: Hymenoptera) from the Ryukyus, Japan. Biogeography 1: 99–106.

[B16] Tomura S (2020) Two species of Chrysidoidea collected by light trap at Takanosu-yama, Fukuoka, Japan. Pulex (99): 824–825. [in Japanese]

[B17] Xu Z-F He J-H Terayama M (2003) The genus *Nipponosega* Kurzenko et Lelej, 1994 firstly recorded from China, with a new species description (Hymenoptera, Chrysididae, Amiseginae) Bulletin de L’Institut Royal des Sciences Naturelles de Belgique, Entomologie 73: 195–196.

